# 3-year follow-up of half-dose verteporfin photodynamic therapy for central serous chorioretinopathy with OCT-angiography detected choroidal neovascularization

**DOI:** 10.1038/s41598-021-92693-z

**Published:** 2021-06-24

**Authors:** Yu-Chen Hu, Yi-Ling Chen, Yen-Chih Chen, San-Ni Chen

**Affiliations:** 1 Department of Ophthalmology, Changhua Christian Hospital, Changhua, Taiwan, ROC; 2 Department of Ophthalmology, Changhua Christian Hospital Yunlin Branch, Yunlin, Taiwan, ROC; 3 School of Medicine, Chung-Shan Medical University, Taichung, Taiwan, ROC; 4 Department of Optometry, Da-Yeh University, Changhua, Taiwan, ROC; 5 Department of Optometry, Central Taiwan University of Science and Technology, Taichung, Taiwan, ROC

**Keywords:** Diseases, Eye diseases, Retinal diseases

## Abstract

To assess the 3-year outcome of half-dose verteporfin photodynamic therapy (PDT) in central serous chorioretinopathy (CSC) with optical coherence tomography angiography (OCT-A) detected choroidal neovascularization (CNV), we performed a retrospective, interventional study. Patients were divided into 2 groups according to the fluorescein angiography: point source leakage in group 1 and diffuse oozing in group 2. Data were collected from patients including changes of best-corrected visual acuity (BCVA), size of CNV, central macular thickness (CMT), choroidal thickness (CT), reabsorption of subretinal fluid (SRF), sessions of half-dose PDT, and the number of intravitreal injections (IVI) of anti-vascular endothelial growth factor (anti-VEGF). There was a total of 34 eyes in 32 patients included. The mean sessions of half-dose PDT was 1.50 ± 0.75. The mean number of IVI of anti-VEGF was 1.38 ± 3.34. BCVA improved from 0.38 ± 0.33 to 0.20 ± 0.22 (*p* < 0.001). Mean CMT was significantly reduced along with reduced CT and increased size of CNV. SRF was totally reabsorbed in 31 eyes. Patients in group 1 had significant less sessions of PDT and better final BCVA. In conclusion, half-dose PDT treatment was effective for CSC with CNV. Patients with diffuse oozing in FA may fare less well with half-dose PDT.

## Introduction

Central serous chorioretinopathy (CSC) is one of the chorioretinal diseases, characterized by neurosensory retinal detachment and/or retinal pigment epithelial detachment (RPED) at the macular area^1^, which is more commonly seen in the middle-aged population^2^, especially in men^2,3^. The use of corticosteroid^4^, pregnancy^4^, smoking^4^, untreated hypertension^4,5^, psychological stress^5^, gastroesophageal disorders^6^, peptic ulcer^7^, cardiovascular disease^5,8^ had been reported as risk factors of CSC.

In the past, complicated choroidal neovascularization (CNV) was reported typically in eyes with chronic CSC. Recently, CNV was also reported to be present in eyes of acute CSC^9^. In the past, CNV-complicating CSC was mostly diagnosed with fluorescein angiography (FA) and indocyanine green angiography (ICGA)^10^, manifesting as ill-defined leakage in FA; in ICGA, it may present as an abnormal vascular branching network at the early phase and late-staining plaque at the late phase. However, it is challenging to diagnose CNV in the eyes of CSC because of the changes of retinal pigment epithelium (RPE), the presence of pigment epithelial detachment (PED), and ill-defined patterns of hyperfluorescence on FA^10,11^. Since the introduction of optical coherence tomography angiography (OCT-A)^12–15^, the detection of CNV in CSC has become much easier and higher rates of CNV among eyes of CSC have been reported.

To date, there is no standard treatment for CSC complicated with CNV. Intravitreal injection of anti-vascular endothelial growth factor (VEGF)^16^, photodynamic therapy (PDT)^17,18^, and combined therapy^19^ had been used with various efficacies. However, all these reports are before the wide use of OCT-A in the detection of CNV in CSC, and some CNV in CSC may not be detected by the traditional dye imaging modalities^20^. Whether the OCT-A detected CNV, which may be smaller and less chronic in nature than the CNV detected by dye angiography, may behave differently to the treatment is still unknown. In addition, different leakage patterns in FA may also respond differently to treatment. Our previous report has shown that eyes with point source leakage in FA respond better to subthreshold micropulse therapy than eyes with diffuse oozing^21^.

The purpose of this retrospective study is to assess the clinical outcomes of half-dose PDT for the treatment of CSC with CNV detected by routine OCT-A screening, and to compare the difference of the clinical characteristics and treatment response of patients with point source leakage and diffuse dye oozing in FA.

## Results

Thirty-four eyes of 32 patients including 22 males and 10 females were included in this study. The mean age at diagnosis was 51.09 ± 7.24 years. The mean duration of symptoms was 10.07 ± 10.78 months before treatment. The mean follow-up duration were 40.88 ± 12.44 months. Of the 34 eyes, 31 had complete reabsorption of SRF at final follow-up, and the other 3 had decreased, but incomplete resorption of SRF. The mean sessions of half-dose PDT were 1.50 ± 0.75. The mean number of intravitreal injections of anti-VEGF after half-dose PDT is 1.38 ± 3.34. Eleven eyes (Case 1,7,8,9,10,15,18,20,23,27,30) received Aflibercept injection and case 28 received Renibizumab injection. Demographic data of the patients are shown in Table 1.

**Table 1:** Basic demographic data of all patients recruited in this article.

No	Age (y)/Sex /Eye	Initial/Final decimal BCVA	Duration of symptoms (months)	Leakage on FA	Initial/ Final CMT (μm)	Sessions of PDT/No of IVI	Final status of SRF
1	52/M/OD	0.2/0.7	19	Point-source	395/203	1/1	Reabsorbed
2	45/M/OD	0.4/1.0	3	Point-source	363/252	1/0	Reabsorbed
3	49/F/OD	0.9/1.0	14	Point-source	382/232	1/0	Reabsorbed
4	41/M/OS	0.8/1.0	3	Point-source	370/256	1/0	Reabsorbed
5	54/F/OD	0.3/0.7	10	Oozing	296/240	2/0	Reabsorbed
6	49/M/OD	0.8/0.9	6	Point-source	419/227	1/0	Reabsorbed
	49/M/OS	0.7/0.9	6	Point-source	316/247	1/0	Reabsorbed
7	43/M/OS	0.6/0.7	9	Point-source	359/223	2/2	Reabsorbed
8	47/M/OD	0.5/0.5	60	Point-source	265/218	1/1	Reabsorbed
9	58/F/OD	0.8/0.9	4	Oozing	280/233	2/2	Reabsorbed
10	60/M/OS	0.3/0.4	10	Oozing	277/152	3/1	Decreased
11	55/F/OS	0.5/0.8	14	Point-source	386/189	1/0	Reabsorbed
12	46/F/OS	0.5/0.7	6	Oozing	348/222	1/0	Reabsorbed
13	49/M/OS	0.2/1.0	9	Oozing	364/273	1/0	Reabsorbed
14	55/M/OD	0.2/0.3	5	Oozing	472/239	1/0	Reabsorbed
15	61/M/OS	0.3/0.5	8	Point-source	336/220	1/2	Reabsorbed
16	47/M/OD	0.5/0.7	7	Oozing	196/203	1/0	Reabsorbed
17	57/M/OD	0.6/0.4	3	Oozing	339/343	3/0	Reabsorbed
18	53/M/OS	0.02/0.2	7	Oozing	296/248	3/9	Reabsorbed
19	32/M/OS	0.4/0.8	8	Oozing	471/197	3/0	Reabsorbed
20	59/F/OD	0.1/0.2	8	Oozing	355/315	3/17	Decreased
21	45/M/OS	1.0/1.0	13	Point-source	489/264	1/0	Reabsorbed
22	67/F/OS	0.6/0.4	26	Oozing	277/208	1/0	Reabsorbed
23	55/M/OS	0.7/0.6	2.5	Oozing	341/206	2/2	Reabsorbed
24	57/M/OD	0.2/0.7	6	Point-source	451/273	1/0	Reabsorbed
25	37/M/OS	1.0/1.0	9	Point-source	464/270	1/0	Reabsorbed
26	56/M/OD	0.3/0.3	7	Oozing	271/200	1/0	Decreased
	56/M/OS	0.4/0.5	5	Point-source	289/230	1/0	Reabsorbed
27	51/M/OS	0.5/0.8	2	Point-source	388/264	2/2	Reabsorbed
28	56/M/OD	0.8/0.3	30	Oozing	187/174	2/7	Reabsorbed
29	50/M/OD	0.6/0.9	10	Point-source	354/263	1/0	Reabsorbed
30	51/F/OD	0.3/1.0	3	Oozing	313/255	2/1	Reabsorbed
31	52/F/OS	0.5/0.8	4	Point-source	475/155	1/0	Reabsorbed
32	46/F/OS	0.6/0.6	6	Point-source	319/209	1/0	Reabsorbed

*BCVA* best corrected visual acuity, *CMT* central macular thickness(um), *FA* fluoresin angiography, *F* female, *IVI* intravitreal injection, *M* male, *PDT* verteporfin photodynamic therapy, *SRF* subretinal fluid, *y* year-old.

**Figure 1﻿:**
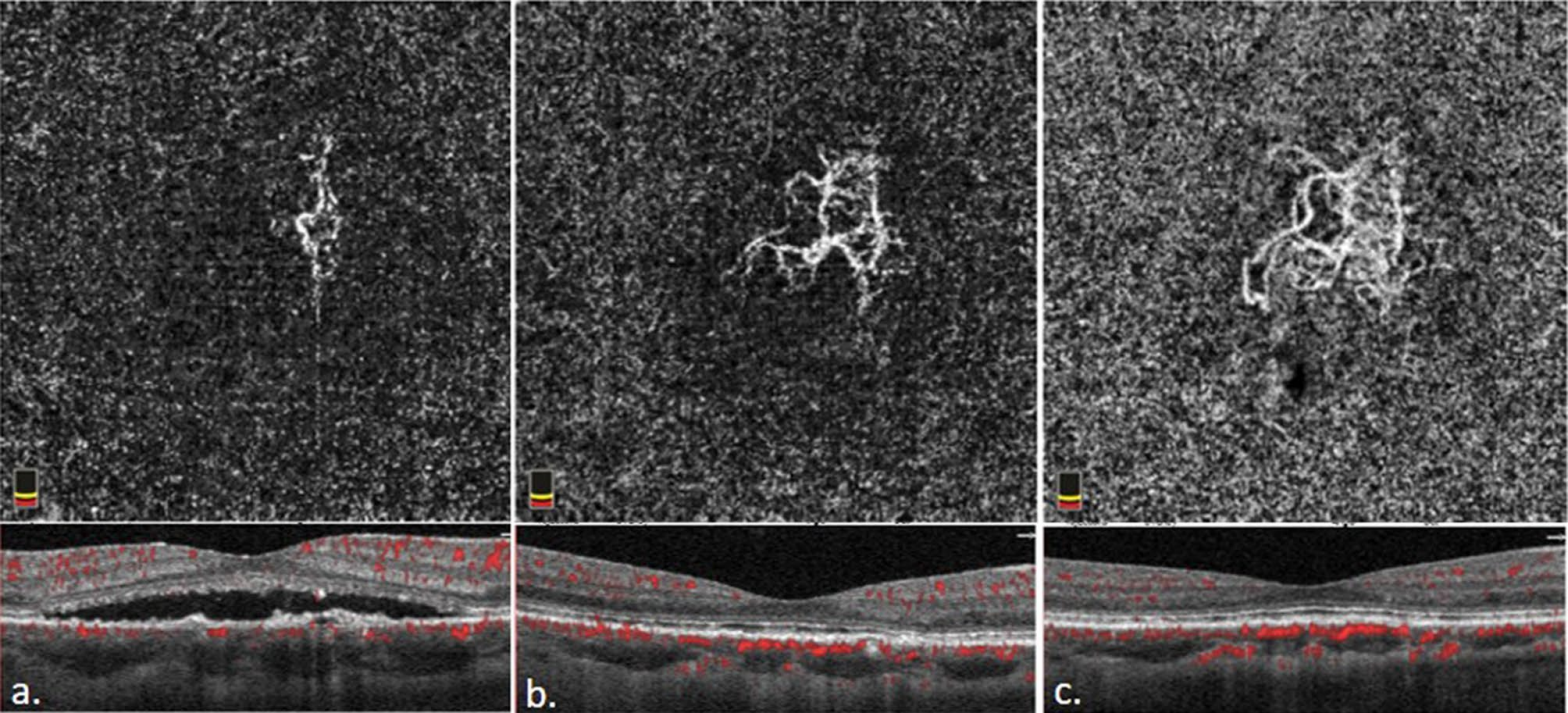
The optical coherence tomography and optical coherence tomography angiography of case 3 before half dose photodynamic therapy (PDT) (**a**). One month later, the subretinal fluid was reabsorbed and choroidal neovascularization (CNV) network was more clearly visible. (**b**) 3-year after PDT (**c**), the size of CNV had enlarged from 0.430 mm^2^ one month after treatment to 0.732 mm^2^ three years after treatment.

**Figure 2:**
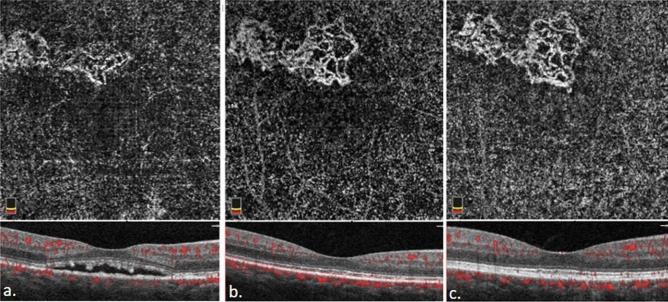
Optical coherence tomography and optical coherence tomography angiography of case 5 before half dose photodynamic therapy (PDT) (**a**). One month after treatment (**b**), the subretinal fluid was reabsorbed completely and choroidal neovascularization (CNV) network was more clearly visible. 3-year after first half-dose PDT(**c**), the size of CNV had enlarged from 0.763 mm^2^ one month after treatment to 1.025 mm^2^ three years after treatment.

There was a significant improvement in BCVA (LogMAR) (*p* < 0.001) and the mean CMT remarkably decreased at the final follow-up (*p* < 0.001) along with a significant decrease in CT (*p* < 0.001). The size of CNV increased significantly (*p* < 0.001) (Table 2), which is nicely shown in Figs. 1 and 2.

**Table 2:** Difference in best corrected visual acuity, central macular thickness, size of choroidal neovascularization, and choroidal thickness between baseline and 3-year follow-up.

	N	Baseline	Final	*p* value
BCVA (LogMAR)	34	0.38 ± 0.33	0.20 ± 0.22	< 0.001*
CMT (µm)	34	350.09 ± 76.25	229.18 ± 35.38	< 0.001*
CNV size^†^ (mm^2^)	34	0.53 ± 0.61	0.99 ± 1.18	< 0.001*
CT (µm)	34	330.89 ± 45.14	245.74 ± 62.96	< 0.001*

Wilcoxon Signed-Rank Test. *p value < 0.05.

*BCVA* best corrected visual acuity, *CMT* central macular thickness, *CNV* choroidal neovascularization, *CT* choroidal thickness, *LogMAR* logarithm of the minimal angle of resolution.

^†^Baseline size of CNV was measured when subretinal fluid totally or subtotally reabsorbed 1 month after photodynamic therapy.

For the types of CNV, all eyes had type 1 CNV noted throughout the course. PCV developed later in one eye (right eye of case 26 in group 2). In addition to SRF, no subretinal hemorrhage or hard exudate was noted throughout the follow-up period. For the treatment previous to half-dose PDT, 2 cases (case 1, 2) had undergone subthreshold micropulse therapy; 1 case had 3 intravitreal injections of bevacizumab (right eye of case 6).

There were 18 eyes from 17 patients in group 1, which had point source leakage in FA. Group 2 comprised 16 eyes in 16 patients which had dye oozing leakage in FA. There was no difference in sex, initial BCVA, size of CNV, and CT between these 2 groups. However, patients in group 1 were younger, had initial thicker CMT, and had fewer sessions of half-dose PDT (Table 3). There were 2 eyes in group 1 that needed one more session of half-dose PDT; in group 2, there were 10 eyes that needed more than 1 session of PDT, of which 5 eyes needed more additional sessions of half-dose PDT. At final follow-up, CMT was reduced and BCVA significantly improved in both groups. CT decreased significantly and the CNV enlarged significantly in both groups. More eyes in group 2 had visible core vessels (*p﻿* = 0.043) and worse final BCVA (*p* = 0.018) compared to those in group 1 (Table 3).

**Table 3:** Differences in age, sex, duration of symptoms, BCVA, CMT, CNV and CT in patients with point source leakage and diffuse oozing.

	Point source leakage (N = 18)	Diffuse oozing (N = 16)	*p* value^b^
	Mean ± S.D	Mean ± S.D	
Age	48.75 ± 6.08	53.44 ± 7.71	0.026*
Sex (M:F)	13 : 4	10 : 6	0.307^c^
Duration of symptoms	10.94 ± 13.06	9.09 ± 7.79	0.798
**BCVA (LogMAR)**			
Baseline	0.28 ± 0.21	0.50 ± 0.40	0.07
Final	0.11 ± 0.11	0.31 ± 0.26	0.018*
*p* value^a^	< 0.001*	0.031*	
**CMT (µm)**			
Baseline	378.89 ± 63.06	317.69 ± 78.54	0.011*
Final	233.06 ± 31.54	226.13 ± 39.63	0.443
*p* value^a^	< 0.001*	< 0.001*	
**CNV (mm**^**2**^**)**			
Baseline	0.36 ± 0.23	0.78 ± 0.88	0.158
Final	0.58 ± 0.46	1.45 ± 1.55	0.384
*p* value^a^	0.025*	< 0.001*	
**Choroid thickness (µm)**			
Baseline	324.48 ± 34.25	338.10 ± 55.22	0.597
Final	234.34 ± 61.21	258.56 ± 64.37	0.695
*p* value^a^	< 0.001*	< 0.001*	
Visibility of Core vessel	3	5	0.043*
Sessions of PDT	1.11 ± 0.32	1.94 ± 0.85	0.006*
Number of IVI	0.44 ± 0.78	2.44 ± 4.72	0.313
BCVA improvement	0.16 ± 0.17	0.19 ± 0.32	0.878
follow up (months)	40.43 ± 14.83	41.38 ± 9.52	0.959

^a^Wilcoxon Signed-Rank Test. *: *p* value < 0.05; ^b^ Mann–Whitney U test. *: *﻿p* value < 0.05; ^c^ Fisher's extract test. *: *p﻿* value < 0.05.

*BCVA* best corrected visual acuity, *CMT* central macular thickness, *CNV* choroidal neovascularization, *logMAR* logarithm of the minimal angle of resolution, *PDT* verteporfin photodynamic therapy, *SRF* subretinal fluid.

^†^Baseline CNV was measured when subretinal fluid was totally or subtotally reabsorbed 1 month after photodynamic therapy.

## Discussion

FA and ICGA were the major imaging modalities for the detection of CNV in CSC in the past. At that time, CSC was reported to be of low incidence^25^ and the presence of CNV was mainly reported to be confined in eyes with chronic CSC^2^. With the advance of imaging modalities and the availability of OCT-A, detection of CNV in CSC became easier and the detection rate was reported to be higher.

Since SRF happens in both CSC and CNV, in eyes with both CSC and CNV, it is difficult to differentiate whether the source of SRF is from the activity of CSC or CNV on the condition that there is no associated hemorrhage, hard exudates or marked macular edema. Both anti-VEGF and PDT have been used in the treatment of CSC with CNV^16–18^. Chhablani et al. reported that by using intravitreal injection of anti-VEGF in eyes with CNV associated with CSC, in which type 1 CNV was present in 43.4% of eyes, the mean changes in BCVA in the number of lines was 1.16 (*﻿p* = 0.23) and the mean number of injection was 4.45 with a mean follow-up duration of 38.3 months^16^. Chan W.M. et al. used full-dose PDT to treat CNV secondary to CSC in a small series of patients (10 eyes), which showed that there is an improvement of 2.4 lines in the mean BCVA after following up for 1 year (*﻿p* = 0.013)^17^. Smretschnig E. et al. used combined therapy of anti-VEGF and half-fluence PDT in eyes of CNV associated with CSC, and demonstrated a non-significant visual improvement (*p﻿* = 0.34) and significant reduction of CMT (*p* = 0.0004) after following up for one year with a mean injection number of 1.8^19^. In the comparative study of CNV associated with CSC by Peiretti et al.^18^, in which more than 50% had PCV, with a mean injection number of 3.44 per year in the anti-VEGF group, and a mean session of 1.56 in the full-dose PDT group, revealed no visual improvement of BCVA in both groups after 1 year of follow-up.

In our study, nineteen eyes (55.9%) received one session of half-dose PDT without recurrence during at least a 3-year follow-up. Fifteen eyes (44.1%) received additional anti-VEGF, half-dose PDT treatment, or both due to persistent or recurrent SRF. Overall, significantly improved BCVA and reduced CMT were obtained, and 91.1% of eyes had complete reabsorption of subretinal fluid with mean 1.50 sessions of PDT and 1.38 intravitreal injections of anti-VEGF.

As compared to previous studies^16–18^, our patients showed a favorable response. The differences may lie in the different characteristics of patients. In our study, all patients had type 1 CNV, and only 1 eye had PCV developed in the later course of follow-up. In addition, all eyes in our study had the CNV detected by routine screening of OCT-A before receiving PDT, which is different from the previous studies, in which FA and ICGA were the major tools used in the detection of CNV^15–17,25^. According to the previous reports, OCT-A is of higher sensitivity in detecting CNV in eyes of CSC^12–14^. Thus, it is possible that the CNV in our series was of shorter duration, less activity and therefore had a better response to the treatment.

In our study, eyes in group 1 had significantly improved BCVA and reduced CMT at final follow-up. Sixteen of the 18 eyes needed only one session of half-dose PDT for SRF to be completely reabsorbed. The treatment response was almost as well as patients with CSC without CNV^25–27^. The eyes in group 2, which needed more sessions of PDT (*p* = 0.006), also revealed significant improvement in CMT and BCVA. Though statistically insignificant, the eyes in group 2 had higher chance to have an intravitreal injection of anti-VEGF. Overall, the treatment response in group 2 was more similar to the previous reports of CSC with CNV^16,18^. The different responses may be attributed to the different sources of SRF in these 2 groups of eyes. Type 1 CNV seldom has point source leakage in FA; CSC, on the other hand, has point source leakage as a typical presentation in FA. Thus, it is reasonable to propose that the eyes in group 1 with point source leakage in our study had the source of SRF from the activity of CSC, rather than the type 1 CNV. In contrast, the eyes that have dye oozing in FA in group 2 may have oozing and SRF either from chronic CSC or CNV, which may explain the different response to half-dose PDT between these 2 groups. In addition to the possible different sources of SRF between these 2 groups, the different characteristics of CNV in the 2 groups may also explain the different responses. In our previous 3-year longitudinal study of CNV in CSC, the proportion of visibility of core vessels in CNV increased after 3 years of follow-up^15^, which implicated that core vessels may more often present in CNV of longer duration. Taking the fact that more core vessels visible along with the older age in group 2, it is possible that eyes in group 2 had more mature CNV, which may respond more poorly to photodynamic therapy.

In the present study, we noted significantly enlarged CNV at the end of follow up, despite the resolution of SRF in most cases, which was in line with our previous study^15^, in which most eyes of CSC and CNV had the CNV quiescent and enlarged during a longitudinal 3-year follow-up. The pathogenesis of CNV secondary to CSC was still unknown. Our previous study has shown that in eyes of chronic CSC, thinner choroidal thickness was noted in eyes with CNV as compared to eyes without CNV^14^. Another report also showed that eyes with flat irregular pigment epithelial detachment with CNV had thinner choriocapillaries as compared to eyes with flat irregular pigment epithelial detachment without CNV^28^. Both studies showed that thinner choroid, and more specifically, choriocapillaries insufficiency and secondary hypoixa, may contribute to the development and enlargement of CNV.

There are some limitations in this study. First, the measurement of the size of CNV may be interfered with the presence of SRF because of the less clear image. Second, the participants in our study were small and the study was retrospective in nature. Third, we were not sure whether eyes with point source leakage may respond well to focal laser or subthreshold micropulse laser therapy or may have spontaneous resorption of SRF without any treatment.

In conclusion, we demonstrated a good mid-term anatomic and visual outcome for patients receiving half-dose PDT therapy for CSC with CNV. Eyes with point source leakage may especially have a better response with visual improvement after one session of PDT. For eyes having diffuse oozing on the FA, there may be higher chance to have further treatment for the regression of the subretinal fluid. Further prospective studies with a larger sample size are necessary to identify the risk factors for recurrence and the validity of half-dose PDT in the management of CSC with CNV and whether combined therapy of anti-VEGF may offer more advantages in eyes with the dye oozing.

## Materials and methods

### Study participants

A retrospective, consecutive, interventional case series of patients over the age of 18 years, diagnosed with CSC and CNV in Changhua Christian Hospital between January 2015 and November 2017 were recruited. All patients had symptoms and subretinal fluid for at least 1 month of duration and received half-dose PDT (3 mg/m^2^ BSA, verteporfin) with a clinical follow-up for at least 3 years. A total of 34 eyes from 32 patients were included. Patients were recorded of age, sex, duration of symptoms, and previous treatment before half-dose PDT. CSC was diagnosed according to the typical findings of FA and ICGA^19,22^, including point source leakage or oozing of RPE, choroidal hyperfluorescence on ICGA, and subretinal fluid (SRF) on optical coherence tomography (OCT). The CNV was identified based on the routine screening by OCT-A in all cases of CSC (Figs. 1 and 2). All eyes had CNV diagnosed before receiving half-dose PDT treatment. The exclusion criteria included: 1. previous treatment for CSC or CNV with PDT; 2. follow-up period less than 3 years; 3. presence of polypoidal choroidal vasculopathy (PCV) lesions by OCT-A or ICGA; 4. other maculopathy on clinical examination. This study adhered to the tenets of the Declaration of Helsinki and was approved by the Institutional Review Board of Changhua Christian Hospital. Informed consent was obtained from each patient. All study participants underwent a complete ophthalmic examination, including best-corrected visual acuity (BCVA), intraocular pressure, slit-lamp, and indirect ophthalmoscopy under pupil dilation. FA and ICGA were taken at baseline. Structural optical coherence tomography (OCT, Heidelberg Spectralis, Heidelberg, Germany) and OCT-A (Optovue, Inc., Fremont, CA, USA) imaging were performed at each visit. For patients with persistent or recurrent SRF, repeated half-dose PDT, intravitreal injection of anti-VEGF, or combined treatment of both were performed after repeated FA and ICGA. Patients were followed up every month, until SRF was completely reabsorbed and later on a 3-month basis.

**Figure 3:**
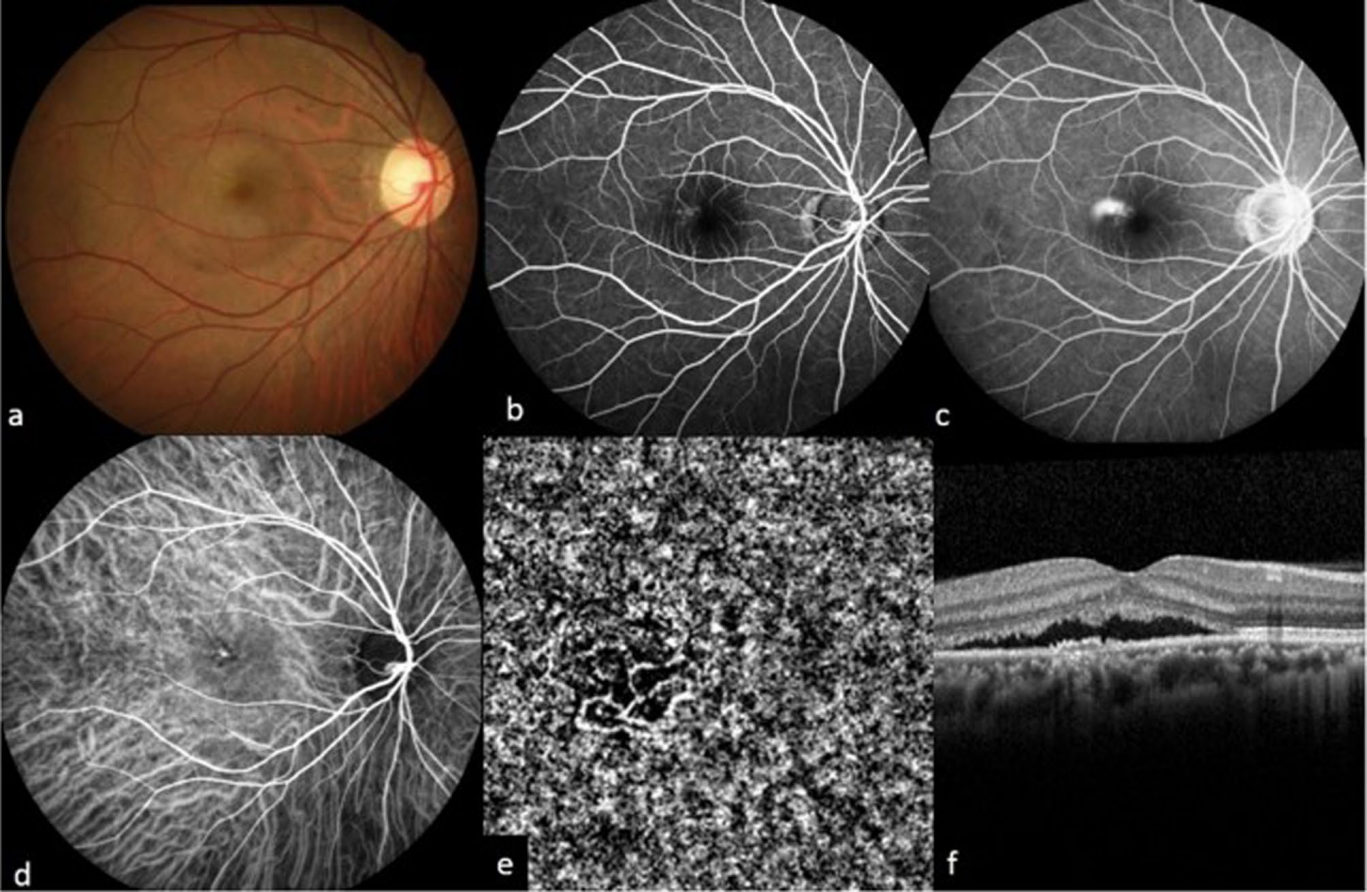
Multimodal imaging of a 45-year old male patient (case 2) in group 1. Fundus photography showed serous detachment at macular area (**a**). Early phase (**b**) and late phase (**c**) fluorescein angiography showed typical point-source leakage and choroidal hyperfluorescence was observed by indocyanine green angiography (**d**). Optical coherence tomography angiography showed choroidal neovascularization (**e**) without visible core vessel and structural oprical coherence tomography showed subretinal fluid accumulation with retinal pigment epithelial irregularity (**f**).

**Figure 4:**
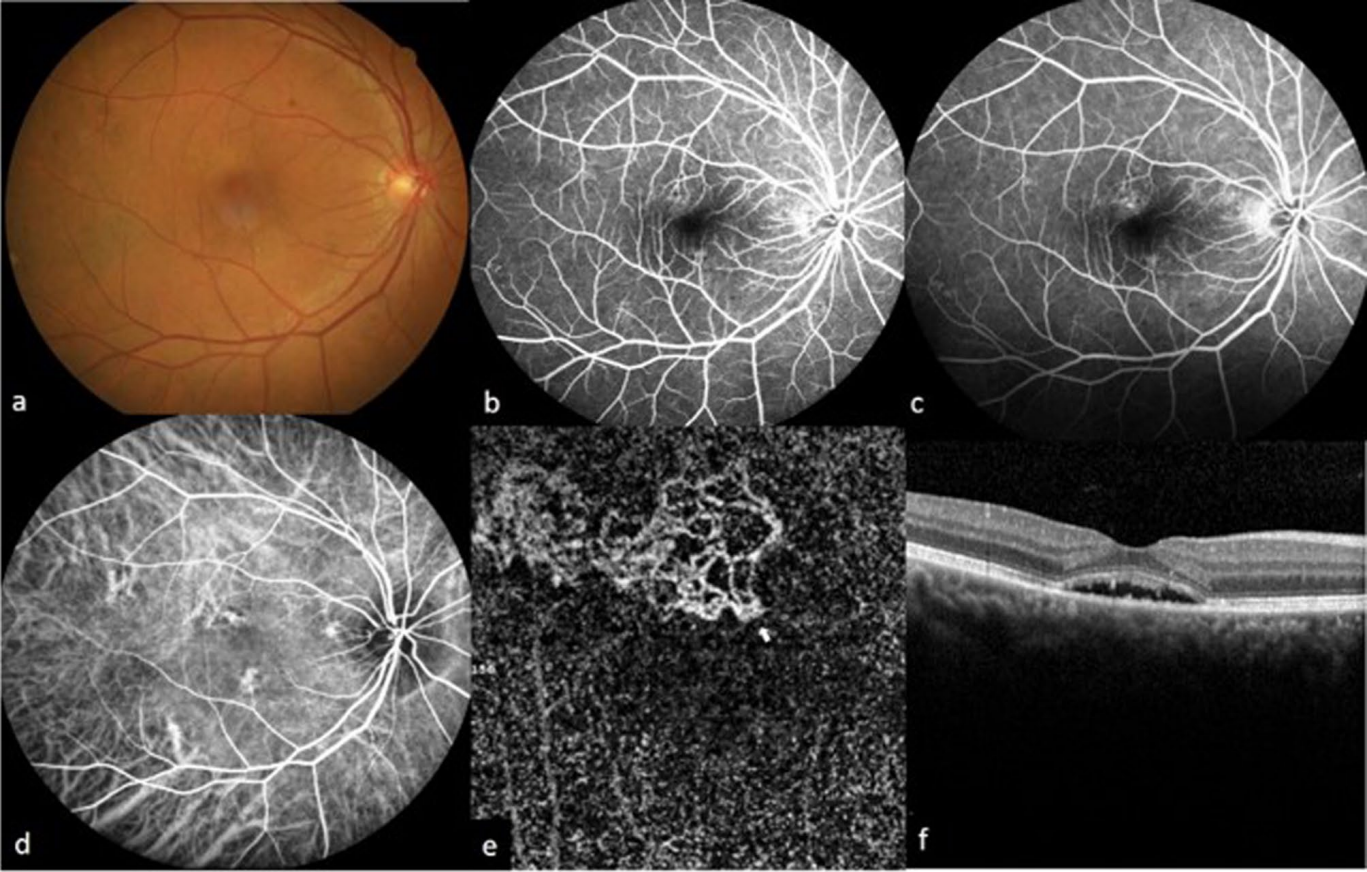
Multimodal imaging of a 54-year old female (case 5) in group 2. The fundus photography shows serous detachment at the macular area (**a**), the hyperfluorescent area demonstrated in early phase (**b**) fluorescein angiography showed oozing pattern at the late phase (**c**), and was demonstrated as choroidal hyperfluorescence in indocyanine green angiography (**d**). Optical coherence tomography angiography showed choroidal neovascularization (**e**) and visible core vessel (arrow). Structural optical coherence tomography revealed subretinal fluid accumulation with retinal pigment epithelial irregularity (**f**).

Patients were divided into 2 groups according to the pattern of leakage in FA: Group 1: patients with point source leakage in FA (Fig. 3), and Group 2: patients with diffuse dye oozing (Fig. 4).

Structural OCT was used to evaluate the presence of SRF, central macular thickness (CMT) and choroidal thickness (CT). CMT was defined as the retinal thickness measured at the area of 500 μm circle centered at the fovea. The distances between the retinal pigment epithelium (RPE) to the chorioscleral interface at the fovea, and at 300 μm, 600 μm nasal and temporal to the fovea were measured and averaged as the CT.

### OCT-A

A 3 mm × 3 mm OCT angiography volume consisting of 304 × 304 A-scans was performed with motion correction^23^. The automated segmentation provided by the OCT-A software was manually customized to 0–30 μm beneath the Bruch membrane to obtain a better visualization of the CNV as previously described^23^.

The image obtained from the OCT-A was analyzed by using Image J software (Image J; U.S. National Institutes of Health, Bethesda, MD, USA) to measure the size of CNV. Visibility of the core vessel according to the criteria described in Carnevali’s study^24^ was evaluated one month after PDT, when the SRF was totally or subtotally reabsorbed and the image was clear enough for evaluation. Size of CNV one month after half dose PDT was used as the initial size of CNV, since the SRF before half dose PDT would obscure the visibility of some area of CNV. All measurements and morphologic analyses were conducted by 2 independent readers (YC Hu, SN Chen). Disagreements over reading were discussed between the readers to reach a final agreement.

### Statistical analysis

Decimal BCVA was converted to the logarithm of the minimal angle of resolution (LogMAR) for calculation. Statistical analysis was performed with the SPSS software package (VER 23; SPSS, Chicago, IL). The Fisher’s exact test was used to compare the difference in gender, the pattern of leakage, and visibility of core vessels between the two groups. Wilcoxon Signed-Rank Test was used to detect the differences in age, BCVA, CMT, size of CNV, and CT at the initial visit and final follow-up. Mann–Whitney U test was used to compare the age, differences of initial and final BCVA, initial and final CMT, initial and final size of CNV, and initial and final CT between the two groups. A *p* value of < 0.05 was considered significant.

## Data Availability

The datasets used and analyzed during the current study are available from the corresponding author on reasonable request.
